# Vibration Propagation on the Skin of the Arm

**DOI:** 10.3390/app9204329

**Published:** 2019-10-15

**Authors:** Valay A. Shah, Maura Casadio, Robert A. Scheidt, Leigh A. Mrotek

**Affiliations:** 1. Department of Biomedical Engineering, Marquette University and Medical College of Wisconsin, Milwaukee, WI 53233, USA;; 2. DIBRIS, University of Genova, 16145 Genova, Italy;; 3. Feinberg School of Medicine, Northwestern University, Chicago, IL 60611, USA; 4. Division of Civil, Mechanical and Manufacturing Innovation, National Science Foundation, Alexandria, VA 22314, USA

**Keywords:** vibration propagation, vibrotactile displays, upper extremity, user feedback

## Abstract

Vibrotactile interfaces are an inexpensive and non-invasive way to provide performance feedback to body-machine interface users. Interfaces for the upper extremity have utilized a multi-channel approach using an array of vibration motors placed on the upper extremity. However, for successful perception of multi-channel vibrotactile feedback on the arm, we need to account for vibration propagation across the skin. If two stimuli are delivered within a small distance, mechanical propagation of vibration can lead to inaccurate perception of the distinct vibrotactile stimuli. This study sought to characterize vibration propagation across the hairy skin of the forearm. We characterized vibration propagation by measuring accelerations at various distances from a source vibration of variable intensities (100–240 Hz). Our results showed that acceleration from the source vibration was present at a distance of 4 cm at intensities >150 Hz. At distances greater than 8 cm from the source, accelerations were reduced to values substantially below vibrotactile discrimination thresholds for all vibration intensities. We conclude that in future applications of vibrotactile interfaces, stimulation sites should be separated by a distance of at least 8 cm to avoid potential interference in vibration perception caused by propagating vibrations.

## 1. Introduction

Four types of tactile mechanoreceptors mediate most of the sensation in human skin: Merkel’s disks, Meissner’s corpuscles (MCs), Ruffini endings, and Pacinian corpuscles (PCs) [1–3]. These mechanoreceptors allow for various haptic sensations such as touch and pressure by Merkel’s disks, skin stretch by Ruffini endings, and vibration by Meissner’s and Pacinian corpuscles [1,4,5]. Haptic perception (touch and vibration) has been studied widely, leading to development of body-machine interfaces (BMI; [6–23]) that can stimulate the skin electrically [7], pneumatically [8], or tactilely to provide performance feedback to users [9–11]. Tactile interfaces are by far the most popular, as they are relatively inexpensive to construct, non-invasive, and can be implemented at various locations on the body where skin sensation remains intact. Tactile stimulation can be implemented using vibrating elements [12–18], pressure [19,20], or skin stretch [21–23]. Interfaces that use vibrotactile stimulations target Meissner’s corpuscles by delivering low-frequency stimulations (5–60 Hz) or Pacinian corpuscles with higher frequency stimulations (60–400 Hz) [2,5,24,25]. With this wide bandwidth of stimulation frequencies available, vibrotactile interfaces can provide a large range of performance feedback information to the user. Our long-term goal is to advance the development of inexpensive and non-invasive BMIs that use vibrotactile interfaces attached to the arm to provide performance feedback to users. Many of these interfaces rely on a multi-channel setup that often use the 2-point touch discrimination threshold (2-TDT) to determine the distance between two stimulation sites. However, this distance may not accurately represent the physical space needed between two vibrotactile stimuli because touch and vibration activate different mechanoreceptors (i.e., Merkel’s disks and MCs/PCs, respectively).

The 2-point distance for vibrotactile stimuli applied to the hand and fingers has been previously investigated [26,27]. Perez et al. found that the 2-point distance on the fingertip for high-frequency stimulations (500 Hz, using piezoceramic vibrating pegs) was more than two-fold higher, at 0.5 cm, than the 2-point distance for low-frequency stimulations (25 Hz) at 0.2 cm [26]. Tannan et al. found that at low-frequency stimulations (25 Hz, using a single probe tip) 2-point distance for the hand dorsum was approximately 0.5 cm [27]. Comparing the two studies, one can see that the 2-point distance changes with body location and vibration frequency. However, results of those studies are difficult to generalize to the skin of the forearm due to differences in mechanoreceptor densities between the glabrous skin of the hand and the hairy skin of the arm [3].

Cipriani et al. used rotating mass vibration motors to investigate the perception of relatively high-frequency vibrotactile stimuli (122–156 Hz) on the volar forearm using three motors, spaced 3 cm apart [13]. Cipriani et al. found that errors in spatial discrimination of vibrotactile stimuli were greater when stimuli were delivered by two motors spaced 3 cm apart compared to motors spaced 6 cm apart. Cholewiak and Collins [28] used inter-motor distances of 2.5 cm on the arm and found vibration localization accuracy as low as 46% (i.e., people were inaccurate in localizing vibration stimuli when the inter-stimulus distance was small). Cholewiak and Collins reported that the localization accuracy increased to 86% when the inter-stimulus site distance was increased to 5 cm. They concluded that interactions between the mechanical and physiological properties of the skin produced interference in vibrotactile localization. It is possible that mechanical propagation of vibration stimuli along the skin can negatively impact vibration perception as mechanoreceptors in the skin adjacent to the site of the vibration may also respond to the stimulus. Thus, space between vibration sites must be increased to reduce interference in vibration perception caused by propagation of vibration stimuli on the skin.

To understand the propagation of vibration, Sofia and Jones [29] measured surface wave propagation of vibrotactile stimuli on the volar forearm using rotating mass motors at ~100 Hz. These vibrotactile stimuli showed the propagation of vibration to a distance of about 2.5 cm from the source of the vibration. If there is interference between two vibration stimuli due to mechanical propagation on the surface of the skin, then the perception of these vibration stimuli will be inaccurate (c.f., Cipriani et al. [13], Oakley et al. [16], and Cholewiak and Collins [28]). However, Sofia and Jones [29] only studied vibration propagation at a vibration frequency of approximately 100 Hz, so how vibration propagates on the skin of the arm during higher frequency vibrations (>100 Hz) remains to be further investigated.

In this exploratory study, we sought to characterize the propagation of vibrotactile stimuli at multiple intensities delivered to the forearm (i.e., between 100–240 Hz). We classified vibration propagation by measuring acceleration across the skin of the arm at various distances from a source vibration of varying intensities. We analyzed changes in acceleration to determine the extent and frequency-dependence of propagation across the human arm. We expect the results will enhance the development of inexpensive BMIs and improve the perception of vibrotactile stimuli in multi-channel, high intensity vibrotactile feedback systems, such as those utilized for hand position feedback for survivors of stroke [18], grip force feedback for upper extremity amputees [17], or to reduce visual attention in people with spinal cord injury [12].

## 2. Materials and Methods

### 2.1. Participants

Six healthy participants (4 females) ranging in age from 19–62 years volunteered to participate in this study. Participants with no known cognitive or sensorimotor deficits of the arm were recruited from the Marquette University community. All participants provided written informed consent to the experimental procedures, which were approved by a local Institutional Review Board in accord with the 1964 Declaration of Helsinki.

### 2.2. General Setup

**Figure 1.**
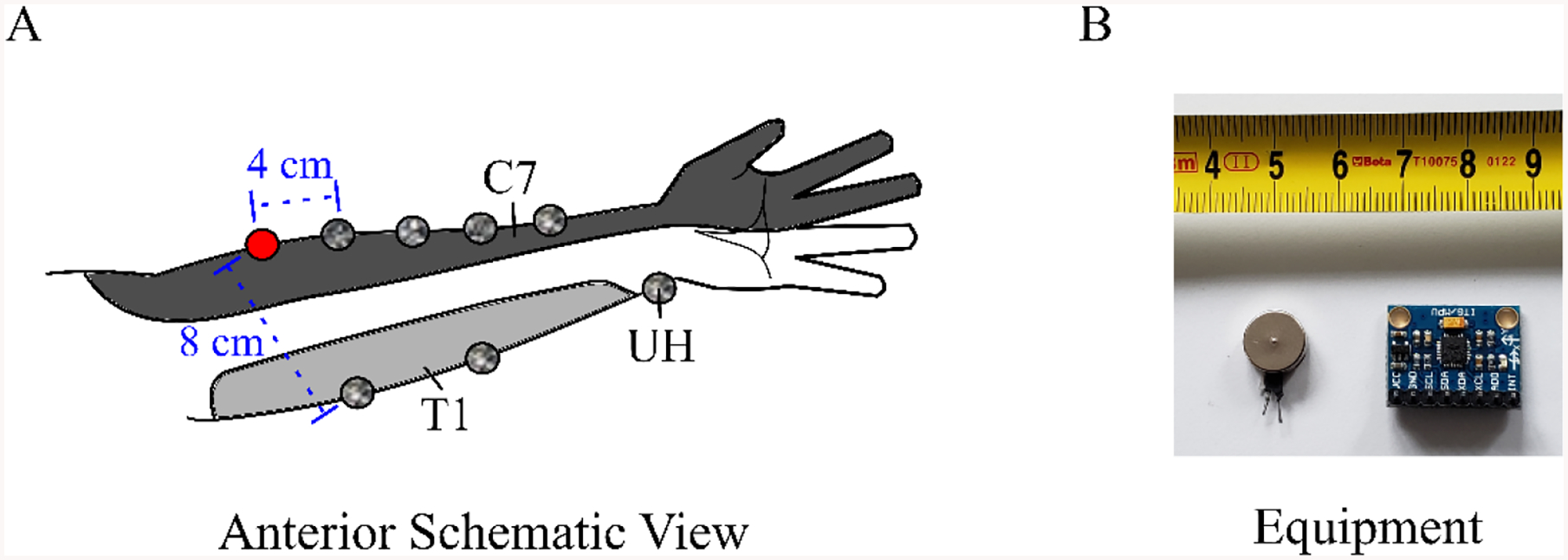
(**A**) Anterior view of the arm. *Red marker* indicates the source vibration. *Gray markers* indicate the locations of acceleration measurements. Example distances from the source are shown as 4 cm in dermatome C7 and 8 cm in T1. (**B**) Equipment. A 10 mm vibration motor next to the MPU-6050 accelerometer mounted on a breakout board.

Each participant completed a single experimental session lasting approximately 30 min. Participants were seated in an armchair with the right hand and arm relaxed on a table, supported by 1-inch thick foam pads. The arm was oriented to have 60 degrees of flexion at the elbow, 15 degrees of shoulder flexion, 0 degrees of shoulder ab/adduction, and the forearm was supinated. Several anthropometric variables were measured: arm circumference (Figure 1: at each gray marker in dermatome C7 in), forearm length (from the lateral epicondyle of the humerus to the radial styloid process while the arm is supinated), and 2-point discrimination distances at the source vibration (Figure 1: red marker); see Appendix A (Table A2). One 10 mm eccentric rotating mass (ERM) motor was used to deliver vibration stimuli (Precision Microdrives Ltd., London, UK, Model # 310–117). These motors have an operational frequency range of approximately 60–250 Hz, coupled to an amplitude range of 0.5–2.4 G [30]. For simplicity, we will refer to vibration intensity throughout this document in terms of frequency because the frequency and amplitude of vibration covary for these ERM motors.

The vibration motor was powered and controlled using custom drive circuitry that was interfaced with a portable laptop computer running a custom script within MATLAB R2017a computing environment (MathWorks Inc, Natick, Massachusetts, USA). The input voltage to the motor was provided through a Pulse Width Modulation signal. Vibration propagation was measured using an InvenSense MPU-6050 (TDK Corp., San Jose, California, USA) 3-axis accelerometer with 16-bit resolution, a full-scale range set to ±2 G, a sampling rate of 1 kHz, and a digital lowpass filter implementing a lowpass cutoff frequency of 260 Hz [31]. The accelerometer was interfaced with the laptop computer using I^2^C communication protocol.

### 2.3. Vibration Propagation Measurement

An ERM vibration motor was attached to the arm on dermatome C7 via Transpore medical tape (3M Inc). The motor was placed approximately 4 cm distal from the later epicondyle of the humerus. The accelerometer was similarly attached to the arm, with the Z-axis perpendicular to the arm and the Y-axis oriented along the lateral forearm. Measurements of vibration propagation were recorded at 7 different locations: at distances of 4, 8, 12, and 16 cm from the vibration motor along the lateral forearm (within dermatome C7); at 8 and 16 cm from the vibration motor along the medial forearm (dermatome T1); and on the ulnar head (UH, dermatome C8). Figure 1 shows the placement of the vibration motor (red marker) and the locations where acceleration measurements were taken (gray markers).

### 2.4. Vibration Stimuli

A total of 12 vibration intensities were tested at each location, with drive voltages ranging from 0.98 V (~100 Hz) up to 3.35 V (~240 Hz). Appendix A shows vibration characteristics for the 12 vibrotactile stimuli (Table A1). The 12 vibration intensities were delivered consecutively to the same location, starting from the lowest intensity and ending with the highest intensity. Each vibration intensity was delivered to the testing location for 1000 ms and the interval between each vibration intensity was 1000 ms. An initial motor drive pulse of 5 ms at 5 V was used to overcome the inertial effects of the ERM motor.

### 2.5. Data Analysis

Measured accelerations along the X, Y, and Z axes of the accelerometer were used to compute the total acceleration [i.e., the Euclidean norm; Equation (1)]:

(1) $$a=\sqrt{\left(a_{x}{}^{2}+a_{y}{}^{2}+a_{z}{}^{2}\right)}$$

We compensated for gravity and variations in accelerometer orientation at each of the different measurement locations by subtracting the acceleration value recorded with the motor turned off. Gravity-adjusted acceleration values reported in the Results and Appendix A were computed from the last 400 ms of stimulation (i.e., well after steady-state vibration was reached).

### 2.6. Statistical Testing

To characterize vibration propagation and the extent to which it attenuates with distance from the source, we used Bonferroni-corrected, one-tailed t-test to compare the acceleration at each measurement location to 0 G. We also used Bonferroni-corrected, one-tailed t-test to compare the acceleration at each measurement location to the vibrotactile intensity discrimination threshold defined in previously published work [32]. This analysis sought to infer the extent to which the propagation of vibratory stimuli could alter vibrotactile perception at each measurement distance. Finally, we used Pearson’s correlation coefficient to determine the extent to which changes in the measured acceleration depend on participant anthropometrics, distance from the source vibration, and source vibration intensity. All analyses were performed with SPSS Statistics 24 (IBM Corp). Statistical significance was set at a family-wise error rate of α = 0.05.

## 3. Results

**Figure 2.**
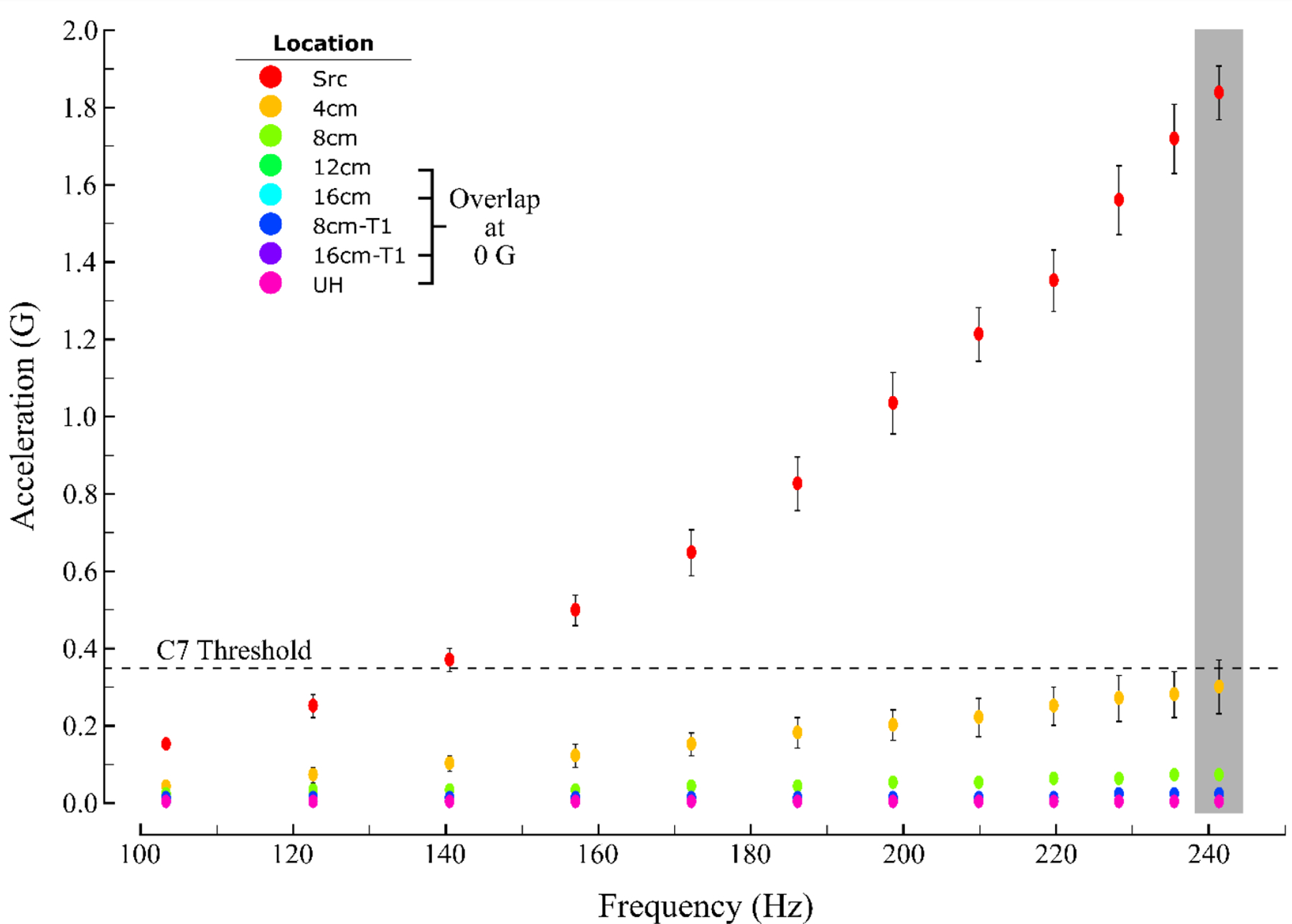
Mean acceleration (across participants) at different distances from a source vibration at various frequencies. The *black dashed horizontal line* shows the amplitude value of the vibrotactile intensity discrimination threshold for dermatome C7. The *gray vertical bar* marks the highest intensity vibration (241 Hz) acceleration values. Error bars show SEM.

This study used an ERM vibration motor and accelerometers to quantify vibration propagation along the human arm. Acceleration on the arm of each participant was recorded during 12 different intensities of vibrotactile stimuli, at seven different measurement locations. As expected, measured accelerations increased as vibration intensity increased, and they consistently decreased as distance from the source increased. Figure 2 shows the measured acceleration values at the seven measurement locations for each tested vibration intensity.

### 3.1. Measured Acceleration as a Function of Source Intensity and Distance

At a distance of 4 cm from the source in dermatome C7, acceleration decreased by 73.3% on average at a vibration intensity of ~100 Hz and by 83.8% at ~240 Hz. At a distance of 8 cm in dermatome C7, acceleration decreased by 86.7% on average compared to the source at ~100 Hz, and by 96.2% at ~240 Hz. At distances of 12 cm and 16 cm in dermatome C7, acceleration at all tested vibration intensities decreased to ~0 G (i.e., less than the bit resolution of the accelerometer: 6.1 × 10^−5^ G). In dermatome T1, acceleration was also negligible across all vibration frequencies at a distance of 8 cm (a maximum 0.02 G at > 230 Hz, a reduction of 99.2%) and decreased to 0 G at 16 cm for all vibration intensities. At the UH (i.e., a distance exceeding 18 cm from the source vibration in all participants), acceleration decreased to 0 G at all tested vibration intensities.

Compared to the ideal “no-propagation” value of 0 G, the Bonferroni-corrected, one-tailed t-test revealed significant differences between the measured accelerations at all vibration intensities for the measured distances of 4 and 8 cm in dermatome C7, and 8 cm in dermatome T1 (p_corrected_ < 0.05 in each case). There were no significant differences at 12 and 16 cm in dermatome C7, at 16 cm in dermatome T1, and at the UH (p_corrected_ > 0.05 in each case).

To determine the extent to which the vibration propagation might interfere with vibration perception, we compared accelerations measured at each location to the amplitude of the vibrotactile intensity discrimination threshold in either dermatome C7 (~0.35 G), dermatome T1 (~0.50 G), or the UH (~0.40 G), respectively (c.f., [32]). In dermatome C7, the measured acceleration at 4 cm was not significantly lower than 0.35 G for intensities greater than 156 Hz (p_corrected_ > 0.05). This result was not a statistical artifact because the tests still showed non-significant differences for frequencies greater than 210 Hz when Bonferroni-correction was removed. Measured accelerations were significantly lower than the C7 discrimination threshold at all vibration intensities at distances of 8, 12, and 16 cm (p_corrected_ < 0.05 in all cases). Measured accelerations were also lower than their respective discrimination thresholds at 8 and 16 cm in dermatome T1 and at the UH (p_corrected_ < 0.05 in all cases).

### 3.2. Acceleration Correlates with Distance

We regressed the acceleration data measured in dermatome C7 onto distance from the source vibration and found a negative correlation (r = 0.943, *p* < 0.05; Figure 2: gray shading). As the distance from the source increased, the measured acceleration decreased. By contrast, we found no correlation between measured acceleration and participant arm circumference, weight, or gender (*p* > 0.05 in all cases). Participant anthropometrics showed no influence on vibration propagation.

**Figure 3.**
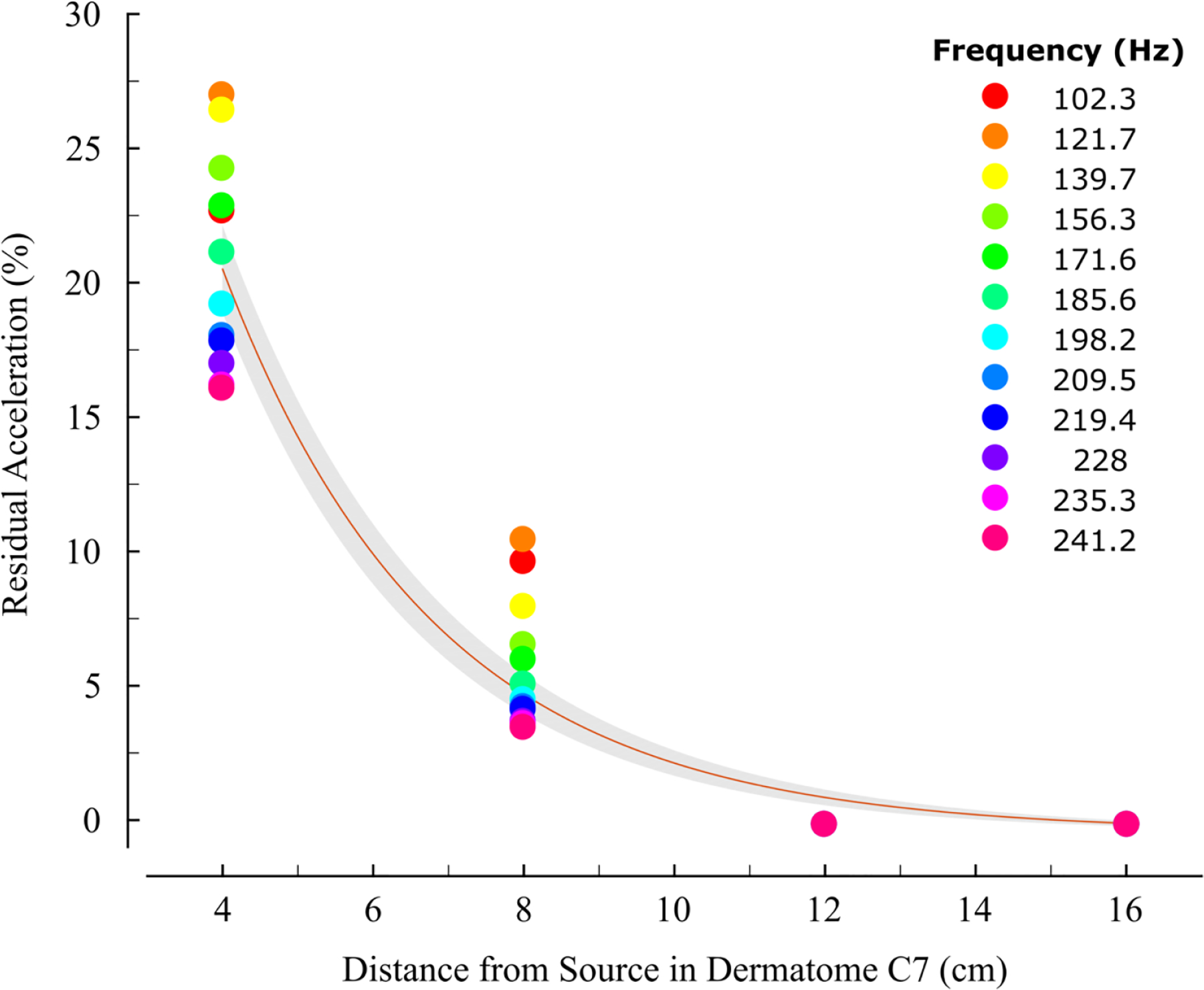
Percentage of acceleration remaining decreases as the distance from the source vibration increases. Data points represent remaining acceleration at the stimulation intensity. *Red line* indicates the non-linear fitted exponential curve, averaged across the 12 vibration intensities. *Gray shaded region* indicates the SEM of the fit.

Figure 3 shows the percent residual acceleration across the four measurement distances in dermatome C7 (i.e., the relative amount of acceleration that remains after accounting for the magnitude of the source vibration). We found that a decaying exponential function reasonably describes the change in acceleration *y* as a function of measurement distance *x* (Equation (2)):

(2) $$y=a*e^{\left(-b*x\right)}$$

where *a* and *b* are constant scaling and rate coefficients, respectively. Across the 12 stimulus intensities, the average goodness of fit was high [R^2^ = 0.927 ± 0.051 (mean ± SD); *a* = 87.66 ± 9.97; *b* = 0.360 ± 0.041]. The standard error of the non-linear model estimate was 1.29% at 4 cm, 0.84% at 8 cm, 0.45% at 12 cm, and 0.12% at 16 cm (Figure 3: gray shading).

Finally, we regressed the percent residual acceleration at 4 cm and 8 cm onto source vibration intensity and found a significant correlation at both locations (4 cm: r = 0.911, *p* < 0.05; 8 cm: r = 0.991, *p* < 0.05). Because the relative amount of vibration varies as a function of source intensity at 4 and 8 cm—even after normalizing (dividing) by the source intensity—these results show that vibration propagation varies as a function of the source’s frequency, and not just its magnitude.

## 4. Discussion

This study investigated the propagation of vibration within and across dermatomes on the hairy skin of the human arm. We measured acceleration on the surface of the arm at various distances from a source vibration, which applied stimuli of varying intensities. Whereas the measured acceleration was highly correlated to the distance between the source and measurement locations, measured accelerations did not covary with participant anthropometrics. Additionally, propagated vibrations were significantly attenuated by more than 95% at distances greater than 8 cm, both within and across dermatomes. At 4 cm and 8 cm testing locations in dermatome C7, the percentage of residual acceleration varied as a function of source stimulus intensity (frequency) even after accounting for differences in source vibration magnitude. Residual vibrations were lower than the amplitude of the vibrotactile intensity discrimination thresholds [32] at each recording location greater than or equal to 8 cm from the source. Our results confirm and extend the results of Jones and Held [33], who measured vibration propagation on simulated skin (viscoelastic materials with properties similar to pig skin) and found that the vibration stimuli were highly attenuated by 6 cm from the source and were reduced close to 0 m/s^2^ at 8 cm (see their Figure 10 [33]). As we discuss below, our findings have important implications for the design of vibrotactile interfaces intended to convey multiple channels of information for use in bidirectional body-machine interfaces (cf., [12,17,18]).

### 4.1. Mechanisms of Perceptual Interference Between Stimulation Sites

Mechanical interference between two closely-spaced vibratory stimuli can negatively impact vibrotactile perception due to superposition (i.e., two vibration intensities can sum together via constructive or destructive interference to create higher or lower intensity vibrations, respectively). Oakley et al. [16] showed that during a discrimination task, vibration intensity can be perceived higher when three vibration motors provide synchronized (in-phase) stimulation in a small area, compared to when a single vibrating motor was activated at a similar frequency. It is also possible that multiple vibrating motors can produce destructive interference, wherein vibration amplitude is attenuated. This can result in lower perceived vibrotactile intensity [13]. Based on the results of the current study, the confounding effects of mechanical interference can be mitigated by providing sufficient distance between two simultaneously activated sources. An inter-stimulus distance of 8 cm suffices to reduce mechanical interference to levels far below vibrotactile intensity discrimination thresholds previously reported in the literature [32].

Physiological considerations such as the density and distribution of the different types of mechanoreceptive afferents found in the skin also influence perception (c.f., [34,35]). In a non-human primate study, Manfredi et al. [36] investigated surface wave propagation of high-frequency vibration (50–1000 Hz) on the glabrous skin of the primate digit. The investigators found that vibration propagated as far as 6.4 cm away from the source and that the propagation also varied with vibration frequency. In that same study, the investigators modeled the response of Pacinian corpuscles to vibratory stimuli and found that the estimated response (i.e., the number of recruited/activated mechanoreceptors) was almost two-fold larger for a 200 Hz stimulus than for a 20 Hz stimulus. Thus, the somatosensory response to vibrotactile stimuli is location- and frequency-dependent. A comparison of measured accelerations at ~100 Hz vs. ~240 Hz at a distance of 4 cm in our study supports the idea that lower-intensity vibrations likely activate a lower number of mechanoreceptors because the vibration does not propagate as far as for higher-intensity vibrations (Figure 2). Our finding of significant correlations between source vibration intensity and percent residual acceleration at 4 and 8 cm confirms and extends the findings of Manfredi et al. [36], who showed that vibration propagation depends on the vibration frequency.

### 4.2. Implications for Design of Vibrotactile Interfaces

Vibrotactile interfaces designed for BMIs often rely on a multi-channel setup, wherein multiple skin sites are stimulated with various frequencies of vibration, with each site encoding stimuli with different meanings [14,37–39]. Some vibrotactile interfaces use the 2-point touch discrimination threshold (2-TDT) to determine the minimum inter-stimulus distance between two stimuli [13,28,40]. The 2-TDT is defined as the distance needed to confidently distinguish between two simultaneous touch stimuli applied to the skin. For dermatomal regions of the arm and forearm, mean 2-TDT values range from 3.1 cm to 4.5 cm [41]. However, the 2-TDT may not accurately represent the distance needed to distinguish between two simultaneous vibrotactile stimuli because different mechanoreceptors are involved in the perception of touch vs. vibratory stimuli (i.e., Merkel’s disks for touch perception vs. MCs/PCs for vibration perception) (cf., [34]; see also [42]).

As shown in Figure 2, we observed mechanical propagation of vibrotactile stimuli across the hairy skin of the arm at distances up to approximately 8 cm. For high-intensity source stimuli, propagated vibrations could be expected to confound perceptual discrimination within a second stimulation channel applied 4 cm from the source. With an inter-site distance of 8 cm however, the magnitude of propagated vibration is just a small fraction of the vibrotactile discrimination threshold. Thus, vibrotactile interfaces that employ low-cost ERM vibrating motors can avoid potential perceptual errors caused by the propagation of high-intensity vibration stimuli if they ensure a minimum distance of 8 cm between two stimulation sources.

We have employed this kind of low-cost vibrotactile interface to mitigate proprioceptive deficits observed in stroke survivors. We attached a multi-channel feedback interface to the less affected arm, with inter-stimulus distances greater than 8 cm. The interface provided hand position feedback of the more affected arm to the non-moving, less-affected arm [18,43,44]. While the system proved to be effective in improving the accuracy of simple, single-degree-of-freedom movements [45], future work is focused on determining efficacy on multi-degree-of-freedom movements. Note that our system builds upon previous designs, which have utilized vibrotactile feedback to provide grip force feedback for upper extremity amputees [17], and to reduce visual attention needed to make movements in people with spinal cord injury [12]. These vibrotactile interfaces could also be applied to other locations of the body if the tactile sense of the arm is affected by disease or injury. Therefore, future studies should look to investigate vibration propagation on the skin of other body regions such as the chest, back, and legs.

### 4.3. Limitations

A limitation of our study derives from our use of inexpensive, off-the-shelf ERM vibration motors that have an operational bandwidth of 60–250 Hz. This bandwidth is smaller than the bandwidth of vibration perception for hairy skin, which ranges from 5–400 Hz [2,5,24,25]. Thus, we did not assess vibration propagation over the full range of frequencies perceptible by humans. Future studies should look to identify inexpensive vibration motors that have a larger operational bandwidth, thereby investigating propagation also at higher frequencies (e.g., between 250–400 Hz).

## 5. Conclusions

In this study, we measured the propagation of 100–240 Hz vibratory stimuli across the hairy skin of the human forearm. Propagation was well modeled as a decaying exponential function of distance from the source. At a distance of 8 cm, the magnitude of propagated vibration was reduced by at least 95% relative to the source at all tested frequencies, and the intensity of propagated vibration was significantly lower than the vibrotactile discrimination threshold for each dermatome spanning the arm and hand. Additionally, vibration propagation was proportional to the source intensity at both 4 cm and 8 cm. From these results, we conclude that future BMIs that utilize vibrotactile interfaces should maintain an 8 cm separation between vibrotactile stimulation sites to avoid potential misperception of simultaneously applied stimuli.

## Appendix A

The manufacturer’s specifications sheet for the ERM vibration motors provides data for vibration amplitude in terms of acceleration when attached to a “standard” test mass of 100 g. These data are replicated in Table A1. This appendix also contains data of subject anthropometrics (Table A2) and mean acceleration measurements at each location from the source and at each vibration intensity averaged across the six participants (Table A3). These data were used in the correlation analyses reported in the main body of the text.

Note that when the motors were applied to the arm rather than the standard test mass, we observed a decrease in acceleration magnitude at the source location due to the larger mass of the arm. On average, the mass of the arm contributed to a decrease in acceleration by 0.50 ± 0.08 G across the 12 tested vibration intensities at the source vibration (Figure 1: *red marker*).

**Table A1.** Vibration parameters as the related input voltage. Frequency and amplitude are reported from the manufacturer specification sheet [30].

**Bit Value (Bits)**	50	60	70	80	90	100	110	120	130	140	150	160
**Duty Cycle (%)**	19.7	23.6	27.6	31.5	35.4	39.4	43.3	47.2	51.2	55.1	59.1	63.0
**Input (V)**	0.98	1.18	1.37	1.57	1.77	1.96	2.16	2.36	2.55	2.75	2.95	3.15
**Freq (Hz)**	102.3	121.7	139.7	156.3	171.6	185.6	198.2	209.5	219.4	228.0	235.3	241.2
**Amp (G)**	0.45	0.65	0.85	1.05	1.20	1.40	1.55	1.75	1.90	2.15	2.25	2.35

**Table A2.** Subject demographics showing age, height, weight, arm length, and arm circumferences along the arm.

Subject	Age (yrs)	Height (cm)	Weight (kg)	Sex	Arm length (cm)	Circumference (cm)					2-TDT @ Source (cm)
						*Source*	@ *4cm*	@ *8cm*	@ *12cm*	@ *16cm*	
1	21	178	83.9	m	26.70	27.90	27.90	26.60	24.10	20.30	3.50
2	18	185	70.3	m	27.90	26.70	26.00	26.00	23.20	19.40	3.75
3	31	157	68.0	f	21.60	25.40	25.40	24.10	20.30	17.80	3.50
4	42	157	52.2	f	22.90	22.20	22.20	21.00	17.80	15.60	2.50
5	23	164	45.5	f	24.40	19.90	18.60	17.30	15.20	13.60	4.50
6	62	157	72.6	f	21.60	25.10	24.80	21.00	17.80	15.90	3.75
**Ave**	**32.83**	**166.33**	**65.42**	-	**24.18**	**24.53**	**24.15**	**22.67**	**19.73**	**17.10**	**3.58**
**SD**	**16.70**	**12.26**	**14.11**	-	**2.65**	**2.97**	**3.29**	**3.55**	**3.45**	**2.53**	**0.65**

**Table A3.** Total acceleration (mean ± SEM) at each distance and test vibration intensity across six participants. Data without SEM indicate an SEM of <0.003.

		Measurement Location (Dermatome)								
		*Source (C7)*	*4 cm (C7)*	*8 cm (C7)*	*12 cm (C7)*	*16 cm (C7)*	*8 cm (T1)*	*16 cm (T1)*	*Ulnar head (C8)*	
**Vibration Intensity (Hz)**	*102.3*	0.15 ± 0.01	0.04 ± 0.01	0.02	0.02	0.00	0.01	0.00	0.00	**Acceleration (G)**
	*121.7*	0.25 ± 0.03	0.07 ± 0.02	0.03 ± 0.01	0.02	0.00	0.01	0.00	0.00	
	*139.7*	0.37 ± 0.03	0.10 ± 0.02	0.03 ± 0.01	0.02	0.00	0.01	0.00	0.00	
	*156.3*	0.50 ± 0.04	0.12 ± 0.03	0.03 ± 0.01	0.02	0.00	0.01	0.00	0.00	
	*171.6*	0.65 ± 0.06	0.15 ± 0.03	0.04 ± 0.01	0.02	0.00	0.01	0.00	0.00	
	*185.6*	0.83 ± 0.07	0.18 ± 0.04	0.04 ± 0.01	0.02	0.00	0.01	0.00	0.00	
	*198.2*	1.04 ± 0.08	0.20 ± 0.04	0.05 ± 0.01	0.02	0.00	0.01	0.00	0.00	
	*209.5*	1.22 ± 0.07	0.22 ± 0.05	0.05 ± 0.01	0.02	0.00	0.01	0.00	0.00	
	*219.4*	1.36 ± 0.08	0.25 ± 0.05	0.06 ± 0.01	0.02	0.00	0.01	0.00	0.00	
	*228.0*	1.57 ± 0.09	0.27 ± 0.06	0.06 ± 0.01	0.02	0.01	0.02	0.00	0.00	
	*235.3*	1.73 ± 0.09	0.28 ± 0.06	0.07 ± 0.01	0.02	0.00	0.02	0.00	0.00	
	*241.2*	1.85 ± 0.07	0.30 ± 0.07	0.07 ± 0.01	0.02	0.00	0.02	0.00	0.00	
